# The Early Steps of Molecule-to-Material Conversion in Chemical Vapor Deposition (CVD): A Case Study

**DOI:** 10.3390/molecules26071988

**Published:** 2021-04-01

**Authors:** Davide Barreca, Ettore Fois, Alberto Gasparotto, Chiara Maccato, Mario Oriani, Gloria Tabacchi

**Affiliations:** 1 CNR-ICMATE and INSTM, Department of Chemical Sciences, Padova University, 35131 Padova, Italy; davide.barreca@unipd.it; 2 Department of Science and High Technology, Insubria University and INSTM, 22100 Como, Italy; ettore.fois@uninsubria.it (E.F.); mario.oriani@uninsubria.it (M.O.); 3 Department of Chemical Sciences, Padova University and INSTM, 35131 Padova, Italy; alberto.gasparotto@unipd.it (A.G.); chiara.maccato@unipd.it (C.M.)

**Keywords:** chemical vapor deposition, density functional theory, zinc oxide precursors, transition metal complexes, molecular dynamics simulations, oxide nanomaterials

## Abstract

Transition metal complexes with β-diketonate and diamine ligands are valuable precursors for chemical vapor deposition (CVD) of metal oxide nanomaterials, but the metal-ligand bond dissociation mechanism on the growth surface is not yet clarified in detail. We address this question by density functional theory (DFT) and ab initio molecular dynamics (AIMD) in combination with the Blue Moon (BM) statistical sampling approach. AIMD simulations of the Zn β-diketonate-diamine complex Zn(hfa)_2_TMEDA (hfa = 1,1,1,5,5,5-hexafluoro-2,4-pentanedionate; TMEDA = *N*,*N*,*N′*,*N′*-tetramethylethylenediamine), an amenable precursor for the CVD of ZnO nanosystems, show that rolling diffusion of this precursor at 500 K on a hydroxylated silica slab leads to an octahedral-to-square pyramidal rearrangement of its molecular geometry. The free energy profile of the octahedral-to-square pyramidal conversion indicates that the process barrier (5.8 kcal/mol) is of the order of magnitude of the thermal energy at the operating temperature. The formation of hydrogen bonds with surface hydroxyl groups plays a key role in aiding the dissociation of a Zn-O bond. In the square-pyramidal complex, the Zn center has a free coordination position, which might promote the interaction with incoming reagents on the deposition surface. These results provide a valuable atomistic insight on the molecule-to-material conversion process which, in perspective, might help to tailor by design the first nucleation stages of the target ZnO-based nanostructures.

## 1. Introduction

The control and modulation of nanometer-level structures are of paramount importance in the fabrication of functional materials for advanced applications, encompassing gas sensing, energy, environmental sciences, and biomedical areas [1]. The consequent extensive research efforts have recently triggered the introduction of nanoarchitectonics, a novel paradigm of materials science and technology on the nanoscale [2] involving the combination of nanotechnologies with other specific disciplines to produce systems with emerging functionalities [3]. In this regard, tailoring of structure, composition, and morphology offers unique opportunities for the applications of metal oxide nanomaterials, which are an endless source of functionalities thanks to the multitude of valence states/structures and the widely diversified chemical reactivity that they exhibit [4,5,6,7,8]. The rational design of such systems is directly dependent on the availability of versatile preparation routes enabling their growth onto suitable substrates allowing, in turn, their direct integration into functional devices [9]. In the current tide of nanomaterial preparation routes, chemical vapor deposition (CVD)-related technologies hold a significant promise thanks to their inherent flexibility, adaptability to large-scale processing, and possibility to yield a broad range of material features by a proper choice of the operational conditions. CVD processes are based on the nucleation and growth of the target materials on solid surfaces starting from suitable molecular precursors in the vapor phase, whose features significantly influence the subsequent molecule-to-material conversion [10,11,12,13,14]. Important research progresses in this area are directly dependent on the attainment of additional insights into the involved reactive processes at the molecular scale, that can be successfully achieved by the combination of advanced experimental techniques and computational modeling [11,15,16,17,18].

Over the last decade, we have focused our attention on the extensive investigation of CVD precursors based on first-row transition metal oxides of general formula ML_2_TMEDA, where L is a fluorine-containing β-diketonate moiety (such as 1,1,1,5,5,5-hexafluoro-2,4-pentanedionate, hfa) and TMEDA is *N*,*N*,*N′*,*N′*-tetramethylethylenediamine. The joint use of both ligands yields the full saturation of metal coordination sphere, ultimately resulting in improved stability, volatility, and mass-transport behavior for CVD applications [19,20,21]. In particular, we have evidenced, in the case of the Cu(hfa)_2_TMEDA compound, a “rock-and-roll over a hot floor” motion consisting in a fast diffusion involving a vibrational excitation of metal-ligand bonds, paving the way to the subsequent precursor decomposition to copper oxide (Cu*_x_*O, with *x* = 1, 2) nanomaterials [22]. Such investigations have been partly extended to the homologous Zn(hfa)_2_TMEDA [23] and Fe(hfa)_2_TMEDA [24] complexes, and the outcomes have revealed different high temperature behaviors for the three systems, which might be responsible for diverse decomposition pathways on the growth surface. In spite of these efforts, an open and hot challenge remains a thorough atomistic comprehension of the metal-ligand bond dissociation, related to the first stages of the molecule-to-material conversion in CVD processes. The understanding of this issue is indeed a crucial step towards the ultimate prediction of material properties, which would indeed disclose general concepts to boost the development of novel functional nanosystems.

On the basis of these results, the present research work aims at providing a step forward in the investigation of these issues by combined calculations involving the density functional theory (DFT) coupled with ab initio molecular dynamics (AIMD) [25]. It is worth noticing that, despite the growing interest in understanding via quantum chemical approaches the microscopic details of molecule-to-material conversion in CVD or ALD processes [26,27,28,29,30,31,32,33], most literature data refer to geometry optimization or transition state calculations performed at 0 K. Whereas these studies contribute to shedding light on the binding of the metal center with substrate atoms, the formation of metal-surface linkages for the target CVD precursors, where the metal center is completely surrounded by ligands, requires the dissociation of at least one metal-ligand bond, and remains a key issue to be further investigated.

The use of computational methodologies capable of taking into account thermal motion would be crucial to identifying possible molecular rearrangement events which, in turn, could promote the metal-ligand bond dissociation in the above molecular systems. Indeed, the rolling diffusion of Cu(hfa)_2_TMEDA [22] and Zn(hfa)_2_TMEDA [23] complexes, accompanied by large temperature-induced molecular distortions, suggests that thermal activation processes on the heated substrate surface might be responsible for both metal-ligand bond dissociation and metal center binding to surface atoms. Unfortunately, the mechanisms of these events are not only unknown, but also difficult to be foreseen on the basis of the sole precursors structure. Additionally, the thermal behavior of the precursor on the heated substrate may depend on the nature of the metal center. As an example, AIMD simulation on Fe(hfa)_2_TMEDA at 750 K revealed that the thermally-promoted rolling motion was sufficient to detach TMEDA from Fe(II) as a neutral ligand, leading to two separately physisorbed Fe(hfa)_2_ and TMEDA moieties [24]. On the other hand, the thermal behavior of the homologous Zn complex on the growth surface evidenced a preferential dissociation of one Zn-O bond [23], suggesting that a change of Zn coordination number from six to five could be the initial reaction step. Nonetheless, the simulation of reactive process such as bond dissociation events at finite temperature conditions generally requires statistical sampling techniques, such as the Blue Moon ensemble [34] approach, which have been successfully applied to the gas-phase fragmentation of Cu(hfa)_2_TMEDA [35]. In this perspective, herein we used a combined Blue Moon-AIMD approach to capture at the molecular level the mechanism of the metal-ligand bond breakage in the target molecular systems. This strategy enables to gather insight on the first stages of the precursor fragmentation on the growth surface and, most importantly, to evaluate the process energy barrier.

As a case study, we focus herein our attention on Zn(hfa)_2_TMEDA as a molecular precursor to Zn(II) oxide nanomaterials, in view of the paramount importance of ZnO for a huge variety of technological end-uses encompassing piezoelectric nanogenerators, sensors, lasers and optoelectronic devices, photocatalysts, solar cells, and supercapacitors [36,37,38,39,40,41].

## 2. Methods

Zn(hfa)_2_TMEDA was synthesized following a previously reported procedure [42]. The compound UV-Vis absorption spectrum was recorded on a Cary 5E (Agilent, Santa Clara, CA, USA) dual-beam spectrophotometer, using a 1.25 × 10^−5^ M ethanolic solution and quartz cuvettes with an optical path length of 1 cm.

The behavior of the Zn(hfa)_2_TMEDA complex on the growth surface was modeled in the framework of DFT. The PBE functional [43] with dispersion corrections [44] was employed along with plane wave (PW) basis sets and a PW cut-off of 25 and 200 Ry for wavefunction coefficients and electronic density, respectively. All simulations were performed with periodic boundary conditions.

The experimental conditions required for the deposition of transition metal oxides from Zn(hfa)_2_TMEDA precursors, i.e., oxidizing atmosphere (O_2_ + H_2_O), support heated in the 523–773 K interval [4], imply the formation of hydroxyl groups on the support, hence an outermost hydroxylated SiO_2_ layer. Accordingly, we employed a hydroxylated SiO_2_ slab model as in previous work on this precursor family [22,23,24]. The slab was characterized by 1 nm thickness, 1.7 × 1.7 nm^2^ surface area, and chemical composition Si_36_O_72_·8H_2_O. The simulation periodic box was 1.7 × 1.7 × 2.6 nm^3^. The 2.6 nm height, which included circa 1 nm vacuum, allowed for good decoupling of periodic images along the z directions. The slab contained 2.75 surface hydroxyls per nm^2^, in line with data on hydroxylated silica surfaces [45,46].

All the simulated [Zn(hfa)_2_TMEDA + surface] models contained a total of 185 atoms (132 atoms for the slab + 53 atoms for the complex). The guess geometry of the systems for simulation runs were built by placing the precursor complex in different and randomly selected positions and orientations in the proximity of the slab. From such geometries, structural optimizations were carried out, and the resulting sets of nuclei positions were used to start AIMD simulations [25,47] using the CPMD program (Version 4.1, ©2019–2021 IBM Corp. & MPI für Festkörperforschung Stuttgart, Zürich (CH) & Stuttgart (D)) [47]. To treat the electron-cores interaction, a norm-conserving pseudopotential [48,49,50] was employed for Si, along with ultrasoft pseudopotentials of the Vanderbilt type [51] for all other atoms. All pseudopotentials are available in the CPMD library. A spin multiplicity of 1 was adopted, consistently with the singlet spin state of the isolated Zn complex [23]. This calculation scheme for the electronic structure yielded a good representation of the behavior of molecules adsorbed on oxide surfaces as well as organic-inorganic materials both at 0 K (optimized geometry) and at finite temperature conditions (molecular dynamics simulations) [22,52]. For the AIMD, a 0.121 fs time step was selected, along with a 500 au fictitious mass for the wavefunction coefficients. After equilibration, the trajectory was followed for 20 ps in the NVT canonical ensemble, with 500 K target temperature and Nose–Hoover chain thermostats [53,54].

**Figure 1:**
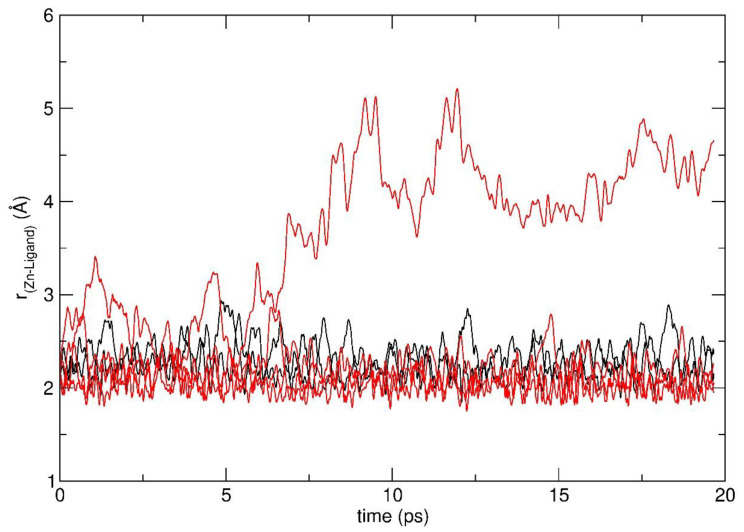
Variation of four Zn-O distances (red solid lines) and two Zn-N distances (black solid line) along the exploratory ab initio molecular dynamics (AIMD) simulation of the octahedral Zn(hfa)_2_TMEDA molecular complex on the hydroxylated silica surface.

The free energy curve for the conversion from hexa- to penta-coordinated Zn in the Zn(hfa)_2_TMEDA complex was computed via a statistical sampling approach known as Blue Moon (BM) ensemble [34], in conjunction with AIMD. As reaction coordinate (or constraint), we selected the distance between the Zn atom and one of the diketonate oxygen atoms (namely, O_2′_, see Figure 1), r = r(Zn-O_2′_). This reaction coordinate was sampled by carrying out, along an interval of 2.72 Å, 18 BM-AIMD simulations. Each simulation was performed by fixing the constraint at the selected value and prolonged until the convergence of the constraint force. The entire set of BM simulations lasted ≈ 130 ps elapsed time. All the constrained simulations were performed by selecting 500 K as the target temperature and by employing Nose–Hoover chain thermostats [53,54].

At the end of the BM-AIMD simulations, selected configurations were extracted from the trajectories corresponding to the initial value of the reaction coordinate (“reactant”: octahedral hexa-coordinated complex) and to the final value of the reaction coordinate (“product”: penta-coordinated complex), as well as from the activated complex region. After simulated annealing, these configurations were subjected to geometry optimization, leading to the 0 K structures of the octahedral complex (Oct@SiO_2_), the pyramidal complex (Pyr@SiO_2_), and the transition state (TS@SiO_2_).

The binding energy (BE) of the Oct and Pyr forms of Zn(hfa)_2_TMEDA on the hydroxylated SiO_2_ surface was calculated using the abovementioned optimized geometries computed for the Zn(hfa)_2_TMEDA/slab system. BEs were computed using the formula:

−BE = E(complex + slab) − E(isolated complex) − E(slab)(1)

where E(complex + slab) is the total energy of Zn(hfa)_2_TMEDA adsorbed on the hydroxylated SiO_2_ surface, and E(complex) and E(slab) are, respectively, the total energies of a single isolated Zn(hfa)_2_TMEDA molecule and of the bare hydroxylated silica slab model, whose geometries have been optimized using the same computational setup and the same periodic simulation box adopted for the (complex + slab) system. With this definition, binding energies are positive.

All the structural optimizations presented in this work were performed by employing a quasi-Newton algorithm [55]. The convergence criterion was set to a maximum of 1 × 10^−4^ atomic units (au) for the maximum value of the gradient per ionic degree of freedom, as done in previous work on these complexes [23]. All the PW calculations were performed with the CPMD code [47].

Hybrid DFT calculations on the Oct and Pyr forms of the isolated Zn(hfa)_2_TMEDA complex in the gas phase were performed using the M06 functional [56], the ECP10-MDF pseudopotential [57] with the correlation-consistent aug-cc-pVDZ-PP basis set for Zn [58], and full double-ζ basis sets augmented with diffuse and polarization functions (D95+(d,p)) [59] for the atoms of the TMEDA and hfa ligands. Basis sets not available in the Gaussian 09 were taken from the basissetexchange.org library [60].

Electronic excitation spectra on the PBE-D2 optimized geometries of the Oct and Pyr forms of the isolated Zn(hfa)_2_TMEDA were computed in implicit solvent ethanol [61] using time-dependent DFT (TD-DFT) with the CAM-B3LYP functional [62] and the standard 6-311++g(2d,p) basis set (available in the Gaussian 09 library [63]), by considering the first 20 lower energy excitations. To obtain the simulated spectra, the obtained TD-DFT values were broadened using a 2-nm width Gaussian function.

All the Gaussian calculations were performed with spin multiplicity = 1 (singlet) using the Gaussian 09 code (Revision B.01, Gaussian, Inc., Wallingford, CT, USA, 2010) [63]. Visualization of molecular structures and supporting movie (Movie S1, Supporting Information) were created with the VMD code (version 1.9.3, TCB Group, University of Illinois, Urbana-Champaign, IL, USA) [64].

## 3. Results

### 3.1. The Square-Pyramidal Zn(hfa)_2_TMEDA Complex

The exploratory AIMD simulation at 500 K shows that the Zn(hfa)_2_TMEDA complex, initially adsorbed with an octahedral geometry, underwent diffusion on the heated substrate via a rolling motion triggered by thermal energy exchange with the substrate, in line with previous AIMD simulations in the 363–750 K temperature range [23]. During this rolling diffusion motion, sketched in Movie S1, the Zn-O and Zn-N bonds showed large oscillations and, in some cases, metal-ligand bonds were transiently broken, thus determining a ligand rearrangement around the metal center (see Movie S1, Supporting Information).

This behavior can be quantitatively described by the time evolution of the metal-ligand distances along the trajectory (Figure 1) and the corresponding statistical values (Table 1). At about 7 ps, one of the Zn-O distances (Zn-O_2′_ from now on) drastically increased up to a maximum value of 5.21 Å, remaining above 3.5 Å for the rest of the simulation, whereas the other metal-ligand distances kept oscillating around typical Zn-O and Zn-N coordination distances, with lower amplitude (see Figure 1 and Table 1). The significant lengthening of the Zn-O_2′_ distance during the simulation was also evidenced by the high value of the average Zn-O_2′_ distance (3.62 Å) and standard deviation (0.91 Å), which is an estimate of the thermal fluctuation of the Zn-O_2′_ distance and was much larger compared to the other Zn-ligand bonds (Table 1). These results indicate that thermal motion on the silica surface had induced a change in zinc coordination number from six to five. Indeed, the Zn-O bond dissociation was followed by a partial detachment of one hfa ligand, leading to the formation of a Zn(hfa)_2_TMEDA characterized by a penta-coordinated Zn center and a square-pyramidal geometry (See Movie S1, Supporting Information).

These data suggest that the square-pyramidal arrangement of the Zn(hfa)_2_TMEDA compound, although never observed at room temperature, might be formed at elevated temperature on a hydroxylated surface.

**Figure 2:**
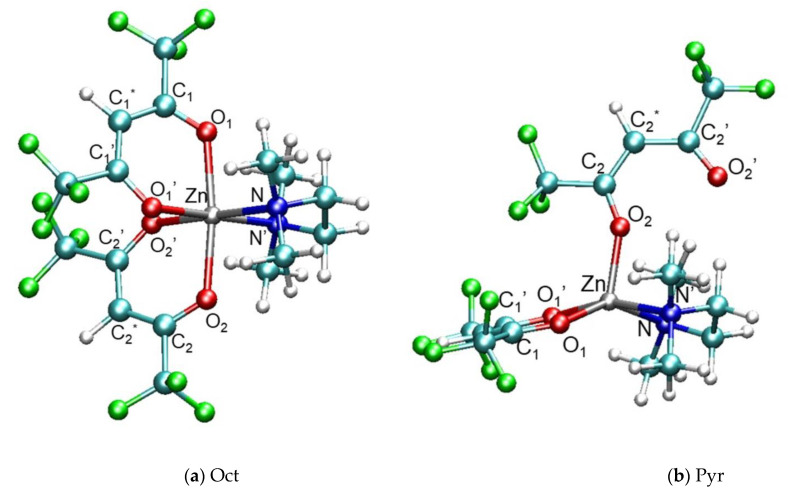
Optimized geometries of the (**a**) octahedral and (**b**) pyramidal forms of the complex Zn(hfa)_2_TMEDA (computed at the M06/D95+(d,p) level of theory). (**a**) Octahedral complex (Oct), which is the minimum energy structure, evidencing the pseudo-octahedral Zn coordination environment. (**b**) Pyramidal complex (Pyr), highlighting the breakage of the bond between the β-diketonate oxygen O_2′_ and the Zn center. Pyr is higher in energy than Oct by 13.06 kcal/mol at the M06/D95+(d,p) level, and by 10.02 kcal/mol at the PBE-D2/PW level. C_1_* and C_2_* indicate the central C atoms of the hfa ligands. Color codes: Zn, grey; F, green; O, red; N, blue; C, cyan; H, white.

To investigate whether the pyramidal geometry of the Zn complex could be stable even in the gas phase at 0 K (in the absence of a surface), we optimized the geometry of an isolated Zn(hfa)_2_TMEDA molecule by using as a guess the final configuration of the 500 K trajectory. The calculation resulted in a stable square-pyramidal geometry, depicted in Figure 2b, in which the pyramid base was characterized by a N’NO_1′_O_1_ dihedral angle of 8.3°. Compared with the standard octahedral geometry of this compound, characterized by a C_2_ symmetry (Figure 2a), the pyramidal structure was 10.02 kcal/mol higher in energy. This result can be easily understood by taking into account that in the pyramidal complex, Zn formed only five bonds with the ligands, i.e., its coordination environment was not fully saturated. Indeed, the minimum energy structure of isolated Zn(hfa)_2_TMEDA, shown in Figure 2a, exhibited six Zn-ligands coordination bonds, and had the C_2_ symmetry documented in previous investigations on β-diketonate-diamine compounds [23,42,65].

On the other hand, the Pyr form was characterized by the detachment of one of the β-diketonate oxygens, O_2′_, which was separated by about 4.3 Å from the metal center (see Table 2). The other oxygen atom of the same diketonate ligand, O_2_, is bound more strongly to Zn, as indicated by the pronounced shortening of the Zn-O_2_ bond distance (see Table 2). In addition, even the bond distances of Zn with the two oxygens (O_1_ and O_1′_) of the second hfa moiety, and with the N atoms of TMEDA, became shorter than in the octahedral structure. These findings suggested a strengthening of metal-ligand interactions as an effect of the loss of the Zn-O_2′_ bond in the Pyr complex. Another interesting consequence of the Zn-O_2′_ bond dissociation was the loss of conjugation of the hfa moiety. The O_2′_-C_2′_ distance elongation was accompanied by the shortening of the O_2_-C_2_ bond: as a result, the C_2_*-C_2′_ and C_2_*-C_2_ bonds became no longer equivalent. Such an effect was indeed significant, especially in comparison with the other hfa ligand, which had very close C-O and C-C bond lenghts.

The DFT results with plane waves (PBE-D2/PW) on the two forms of the complexes agreed quite satisfactorily with calculations at higher level of theory performed with the hybrid DFT functional M06 and D95+(d,p) Gaussian basis sets (M06/D95+(d,p)). In particular, the metal-ligand bond distances of the Oct complex calculated with PBE-D2/PW were slightly higher (on average, by about 1.4%) than the corresponding M06/D95+(d,p) values (Table 2). The same effect was observed even for the Pyr structure: specifically, the PBE-D2/PW metal-ligand bond distances were overestimated by 1.4% with respect to the M06/D95+(d,p) ones. Importantly, the distances of the detached oxygen atom O_2′_ from Zn obtained with the two methodologies differed by only 0.7%, and also the N’NO_1′_O_1_ dihedral angles defining the square-pyramidal geometry were very close (8.3° vs. 9.0° for PBE-D2/PW and M06/D95+(d,p), respectively). Finally, even the diketonate C-C and C-O bond distances, as well as the Zn-centered bond angles computed with the hybrid functional, were fairly well reproduced by the PBE-D2/PW approach for both the Oct and Pyr forms of the complex (see Table 2).

The energy difference between the Pyr and Oct form at the M06/D95+(d,p) level (ΔE = 13.06 kcal/mol) indicated that, in line with the PBE-D2/PW results, the octahedral complex was appreciably more stable than the pyramidal form. Overall, these results could be taken as a validation of our modeling strategy for the finite temperature reactivity of Zn(hfa)_2_TMEDA on the CVD growth surface. As a matter of fact, due to the high computational overhead of statistical sampling approaches on oxide material surfaces [11,52,66,67,68], the simulation of the Zn-ligand bond dissociation process is currently viable at reasonable time scales only through (dispersion-corrected) generalized gradient approximation DFT approaches such as the PBE-D2/PW one.

**Figure 3:**
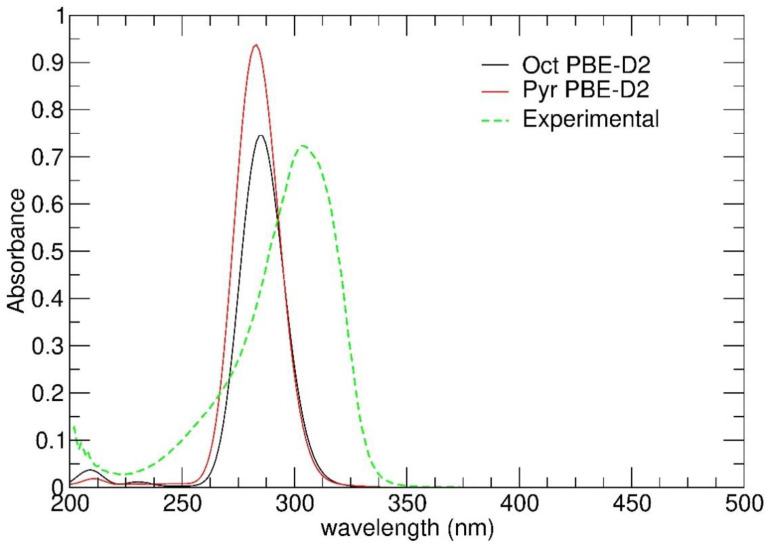
Simulated electronic spectra of the octahedral and pyramidal forms of Zn(hfa)_2_TMEDA along with the experimental spectrum obtained at room temperature in 1.25 × 10^−5^ M ethanol solution.

Before investigating the octahedral-to-pyramidal conversion of Zn(hfa)_2_TMEDA on a hydroxylated silica slab by BM-AIMD simulations, we checked if the structural differences between Oct and Pyr may have consequences also on the electronic structure of the corresponding complexes. To this aim, we computed electronic excitation spectra and compared them with the experimental UV-Vis spectrum of Zn(hfa)_2_TMEDA (octahedral) in ethanol solution (Figure 3). Although the TD-DFT-predicted position of the absorption maximum was shifted towards lower wavelengths, the peak intensity was close to the experimental spectrum, which presented a net UV absorption centered at 303 nm. Overall, the agreement of the Oct simulated spectrum with the experimental one may be considered satisfactory, taking into account that it was obtained from a single configuration—the 0 K minimum energy structure of the complex. Moreover, Figure 3 shows that the two forms (Oct and Pyr) had very close absorption maxima and might not have been experimentally distinguishable. On this basis, we concluded that possible Oct → Pyr isomerization events should not have had appreciable effects on the electronic excitation properties of the Zn(hfa)_2_TMEDA precursor.

**Figure 4:**
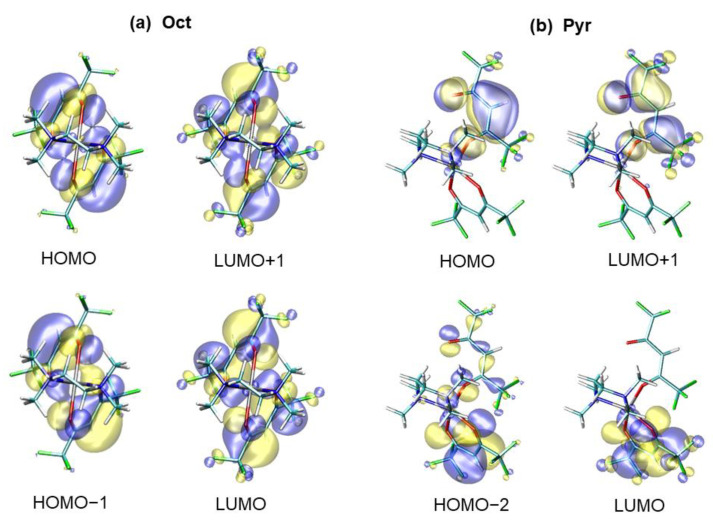
(**a**) Molecular orbitals (MOs) involved in the π–π* ligand-to-ligand electronic transitions for Zn(hfa)_2_TMEDA-Oct in ethanol. The main components of the transition were at λ = 273 nm (oscillator strength = 0.376) (HOMO → LUMO+1 and HOMO−1 → LUMO excitations) and λ = 281 nm (oscillator strength = 0.109) (HOMO → LUMO and HOMO−1 → LUMO+1 excitations). (**b**) MOs involved in the π–π* ligand-to-ligand electronic transitions for Zn(hfa)_2_TMEDA-Pyr in ethanol. Main components at λ = 269 nm (oscillator strength = 0.383) (HOMO*→* LUMO+1 excitation) and λ = 276 nm (oscillator strength = 0.252) (HOMO−2 *→* LUMO excitation). Blue and yellow colors mark positive and negative phases of the MOs, respectively.

Regarding the nature of the electronic transitions, the Oct absorption maximum was mainly due to the HOMO → LUMO+1 and HOMO−1 → LUMO excitations. The four Oct molecular orbitals (MOs) involved in the excitations are shown in Figure 4a. These MOs are spread on both hfa moieties. Whereas HOMO and HOMO−1 had a dominant π-character, LUMO and LUMO+1 were essentially π*. Hence, in the octahedral complex, the occurring transition was ligand-ligand π-π*.

Even in the pyramidal complex, the electronic absorption maximum was mainly ligand-ligand π-π*. However, the relevant Pyr MOs were preferentially localized on a single hfa ligand (Figure 4b). The main transition component was the HOMO → LUMO+1 excitation, involving the π and π* states of the partially detached hfa. The other component was the HOMO−2 → LUMO excitation: the HOMO−2 also contained σ and n-contributions from the oxygen lone pairs, whereas the LUMO was composed by the π*-states of the doubly coordinated hfa. The reason for which the dominant Pyr-Zn(hfa)_2_TMEDA excitation was essentially localized on a single hfa moiety (the mono-coordinated one) with negligible participation of the other hfa ligand could be the symmetry loss upon passing from the Oct (C_2_) to the Pyr (C_1_) geometry.

### 3.2. Free Energy Profile for the Octahedral-to-Pyramidal Conversion on the Hydroxylated Silica Surface

**Figure 5:**
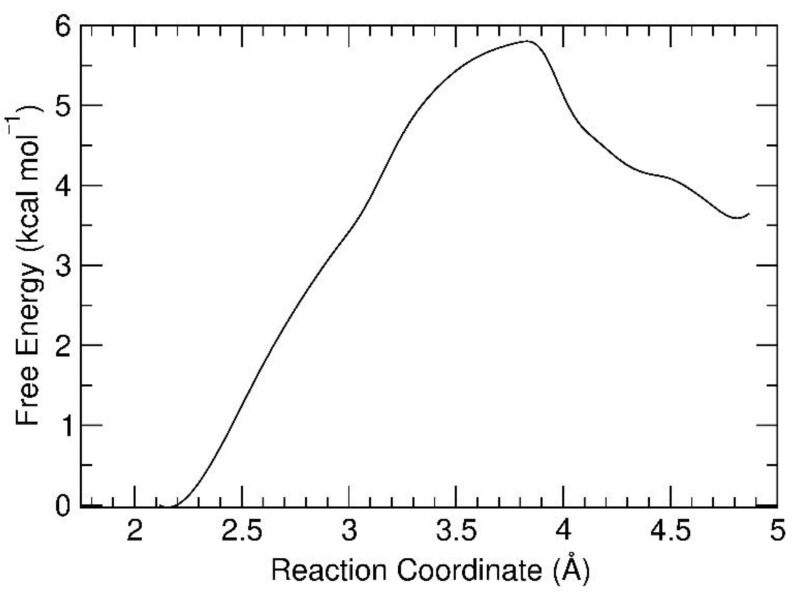
Free energy profile of the octahedral-to-pyramidal conversion of Zn(hfa)_2_TMEDA on the hydroxylated SiO_2_ surface. The reaction coordinate is the Zn-O_2′_ distance.

Having verified the stability of the square-pyramidal Zn(hfa)_2_TMEDA complex both in the gas phase at 0 K and in contact with the silica slab at 500 K, we tackled, using BM-AIMD, the question of how this geometry could be obtained from the octahedral structure under conditions close to the experimental ones. Hence, we positioned the Oct optimized complex on the silica surface and, after equilibration at 500 K, we started the BM sampling from octahedral Zn(hfa)_2_TMEDA stably physisorbed on the slab. As a reaction coordinate, we chose the Zn-O_2′_ bond length, which was progressively elongated from 2.146 Å (a distance typical of the octahedral complex) to 4.869 Å (pyramidal complex) by performing 18 BM-AIMD simulations, each characterized by a fixed Zn-O_2′_ distance (see Methods section). The resulting free energy profile (Figure 5) indicated that the octahedral to pyramidal conversion of the precursor complex was clearly endoergonic, as the free energy difference ΔG of the final product (Pyr@SiO_2_) with respect to the initial state (Oct@SiO_2_) was +3.6 kcal/mol (Table 3). This result might have been expected, since the pyramidal structure was energetically unfavored also in the gas phase. We note, however, that this free energy difference at 500 K was considerably lower than the energy difference between the two forms in the gas phase (10.02 kcal/mol), indicating that thermal activation played a key role in the overall process.

The estimated barrier for the octahedral-to-pyramidal rearrangement amounted to 5.8 kcal/mol, which was comparable to the thermal energy available in the environment (kT at 500 K ≈ 1 kcal/mol). Hence, although the pyramidal geometry was unfavored in the isolated complex at 0 K, this unusual structure became easily accessible if the precursor was in contact with a heated substrate due to the low activation barrier. Interestingly, the BM-AIMD simulations confirmed that the formation of a hydrogen bond between O_2′_ and a surface hydroxyl proton accompanied the generation of the activated complex, contributing thus to the achievement of a low barrier for this process.

The activated complex was formed at a Zn-O_2′_ value of ≈ 3.7 Å, i.e., it was closer to the product side than to the reactant side. Accordingly, the geometry of the complex during the corresponding BM-AIMD trajectory was quite reminiscent of the pyramidal Zn(hfa)_2_TMEDA structure.

The energy difference ΔE between (Pyr@SiO_2_) and (Oct@SiO_2_) and the energy barrier calculated at 0 K are also reported in Table 3. The ΔE value between pyramidal and octahedral geometry (2.23 kcal/mol) was substantially lower than the value for the isolated complexes (10.03 kcal/mol) and confirmed the essential contribution of surface hydroxyls in stabilizing the pyramidal structure via hydrogen bonding. The barrier was found to be 2 kcal/mol higher at 0 K than at 500 K, highlighting thus the vital role of thermal effects in promoting the conversion process.

**Figure 6:**
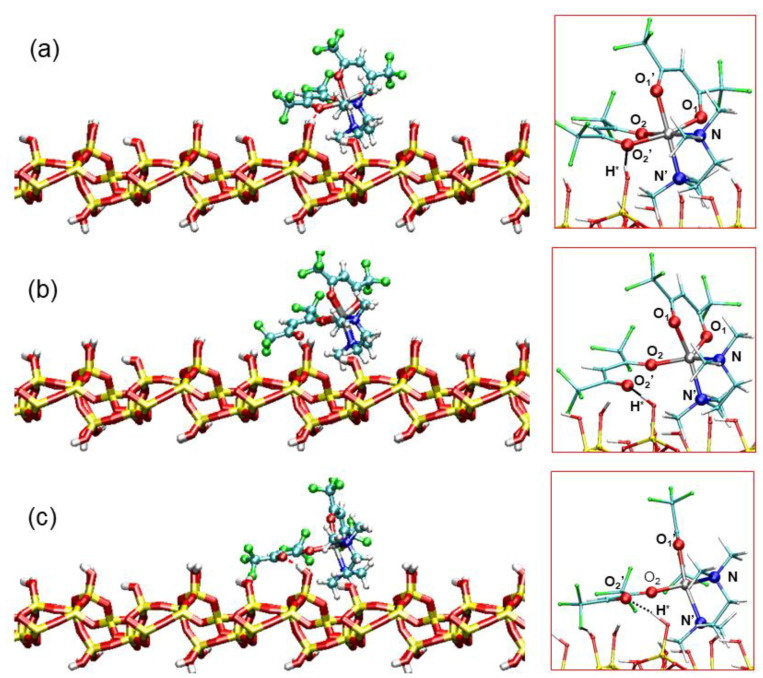
Optimized geometries of the (**a**) octahedral Zn(hfa)_2_TMEDA complex (initial state), (**b**) transition state, and (**c**) pyramidal Zn(hfa)_2_TMEDA (final state) on the hydroxylated silica slab (PBE-D2/PW level of theory). The insets on the right side of the (**a**–**c**) panels highlight the coordination environment of Zn and the O_2′_-H* hydrogen bond. Color codes: Zn, gray; F, green; O, red; N, blue; C, cyan; H, white; Si: yellow. Labels as in Figure 1 and Table 1 and Table 3. The hydrogen bond of the detached hfa oxygen O_2′_ with the surface proton H* is indicated as a dotted line.

Table 4 reports relevant geometrical parameters of the 0 K transition state (TS@SiO_2_), along with those for the optimized structures of (Oct@SiO_2_) and (Pyr@SiO_2_), while graphical representations of these structures can be found in Figure 6. First of all, we noticed the formation of a strong hydrogen bond (1.885 Å) between the O_2′_ oxygen atom (involved in the reaction coordinate) and a surface hydroxyl proton H*. In spite of this interaction, the geometry of physisorbed octahedral Zn(hfa)_2_TMEDA did not show substantial distortions with respect to the isolated Oct complex (Figure 6a). On the other hand, the geometry of the transition state was trigonal bipyramidal, as the Zn-O_2′_ bond was practically broken (Zn-O_2′_ distance being 3.818 Å) and the metal center was 5-coordinated. The N’NO_1′_O_1_ dihedral angle in the transition state was ≈ 55°, indicating that the conversion to the square-pyramidal complex was already at an advanced stage, although not fully completed (Figure 6b). Importantly, the H*-O_2′_ hydrogen bond distance shortened to 1.630 Å, indicating a further strengthening of this interaction, which played a crucial role in stabilizing the transition state. Furthermore, the final state was characterized by the accomplishment of the Zn(hfa)_2_TMEDA square-planar structure (Figure 6c). The N’NO_1′_O_1_ dihedral angle was 23°, indicating that the precursor interaction with the surface led to a pyramidal geometry slightly distorted with respect to the isolated Pyr complex. In addition, in the Pyr@SiO_2_ structure, the distances of both O_2′_ and O_2_ atoms from the metal center were higher than in the isolated precursor (see Table 4). These differences could be attributed to the presence of a very strong hydrogen bond, characterized by a distance (1.586 Å) close to those typical of proton-sharing moieties on oxide surfaces [17,66,69,70,71,72]. Moreover, the complex pyramidal geometry also favored the formation of a hydrogen bond between a surface -OH proton and an F atom of the hfa ligand (F-H distance = 2.002 Å). These interactions counterbalanced the partial detachment of the hfa ligand, thus providing an effective stabilization to the pyramidal complex.

**Figure 7:**
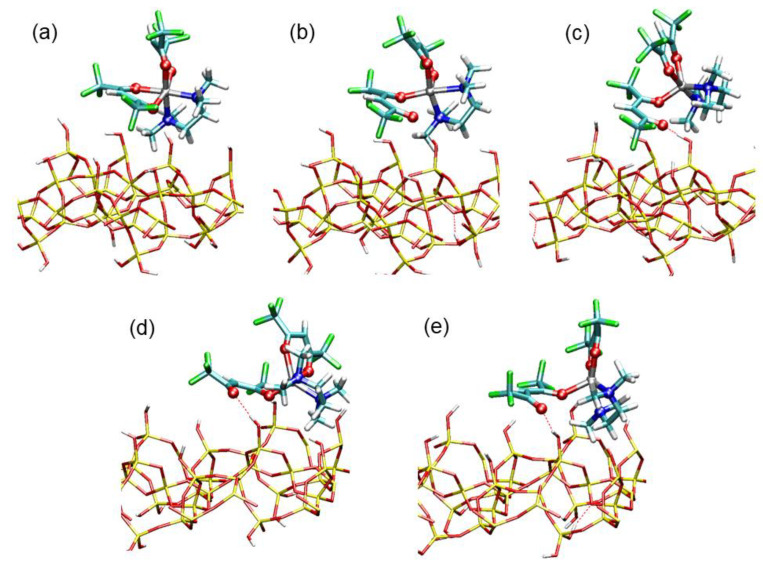
Five snapshots taken from the Blue Moon AIMD (BM-AIMD) simulations corresponding to the following values of the Zn-O_2′_ distance (reaction coordinate): (**a**) Zn-O_2′_ = 2.12 Å, Oct- Zn(hfa)_2_TMEDA; (**b**) Zn-O_2′_ = 3.18 Å, Oct- Zn(hfa)_2_TMEDA; (**c**) Zn-O_2′_ = 3.81 Å, trigonal- bipyramidal Zn(hfa)_2_TMEDA (transition state); (**d**) Zn-O_2′_ = 4.23 Å, Pyr-Zn(hfa)_2_TMEDA; (**e**) Zn-O_2′_ = 4.87 Å, Pyr- Zn(hfa)_2_TMEDA. Color codes: Zn, gray; F, green; O, red; N, blue; C, cyan; H, white; Si: yellow. The hydrogen bond of the detached hfa oxygen O_2′_ with the surface hydroxyl proton H* is indicated as a dotted line.

**Figure 8:**
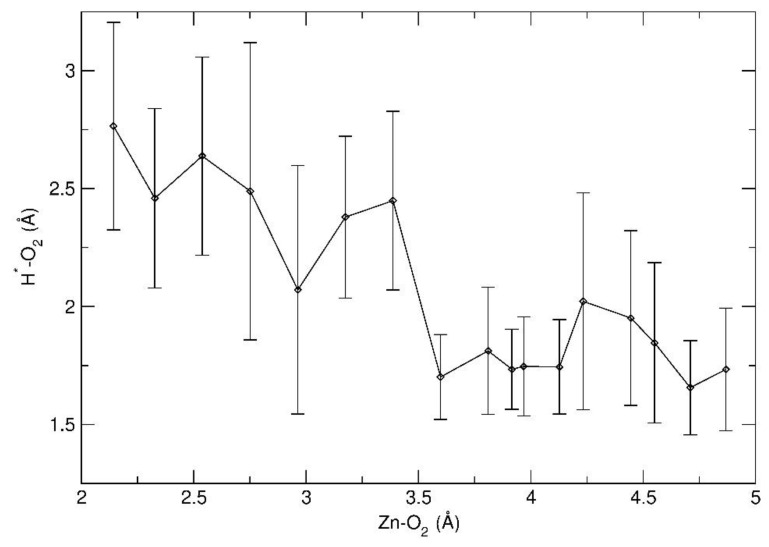
Variation of the average distance between the surface hydroxyl proton H* and the hfa oxygen O_2′_ as a function of the Zn-O_2′_ distance (reaction coordinate). Vertical bars represent the standard deviations, which are an estimate of the amplitude of the thermal oscillations of the O_2′_-H* distance. The black dots correspond to the BM-sampled values of the Zn-O_2′_ distance.

A more detailed insight into the mechanism of the octahedral-to-pyramidal isomerization process was provided by the analysis of structural changes that the complex underwent as a function of the reaction coordinate (i.e., the Zn-O_2′_ distance). Figure 7 shows relevant snapshots taken from the BM-AIMD simulations for increasing values of the Zn-O_2′_ distance. During the first part of the reaction profile (Figure 7a,b), the complex maintained approximately an octahedral geometry, in spite of the significant Zn-O_2′_ bond weakening. Notably, in this part of the reaction, no stable hydrogen bond was formed between O_2′_ and surface hydroxyl proton H*, as evidenced in Figure 8. Indeed, the conversion of the precursor to the pyramidal form started to occur only upon formation of the O_2′_-H* hydrogen bond, corresponding to a Zn-O_2′_ value of 3.6 Å (Figure 8). Figure 7c shows the trigonal bipyramidal structure of the activated complex, which was stabilized by the O_2′_-H* hydrogen bond.

As shown in Figure 8, for larger values of the reaction coordinate, the O_2′_-H* hydrogen bond was always maintained, although with large thermal oscillations. In spite of the high simulation temperature, the average hydrogen bond distances were rather short, indicating a strong hydrogen bond interaction. The latter resulted in a considerable stabilization of the penta-coordinated form of the complex, which could thus evolve to its final square-pyramidal geometry (Figure 7d,e). Hence, the present analysis disclosed the atomistic structural changes of the Zn precursor along the reaction path which showcase the key role of hydrogen bonding with surface hydroxyl groups in the precursor isomerization process.

## 4. Discussion

As a matter of fact, the binding energy of the square-pyramidal Zn(hfa)_2_TMEDA with the surface was 7.81 kcal/mol greater than that obtained for the octahedral Zn(hfa)_2_TMEDA complex (Table 3). Hence, the lower energy of (Oct@SiO_2_) with respect to (Pyr@SiO_2_) could essentially be ascribed to the fact that the octahedral geometry of Zn(hfa)_2_TMEDA was intrinsically energetically favored with respect to the pyramidal structure, since it was characterized by a fully saturated coordination environment, which also implies greater dispersion effects.

The reason why dispersion effects favor the octahedral form of Zn(hfa)_2_TMEDA may be visually appreciated by comparing Figure 6a (Oct@SiO_2_) and Figure 6c (Pyr@SiO_2_). Indeed, the more compact structure of the Oct complex maximized intramolecular dispersion interactions. In a different way, the structure of the pyramidal complex was more prone to undergo hydrogen bonding interactions with the surface atoms. In addition, the pyramidal form exposed a free coordination position of the metal center, which could more efficiently interact with an incoming reagent (e.g., O_2_) on the growth surface. Following this suggestion, an interesting future development of this work might be the study of the interaction of O_2_ with the Zn center in the physisorbed pyramidal complex (Pyr@SiO_2_) and the subsequent oxidation reaction, which would, however, require the use of more computationally expensive spin-polarized calculations.

An unexpected result emerging from this study is the great relevance of the dispersion interactions in the complex-surface binding energy. Indeed, the binding energy of octahedral Zn(hfa)_2_TMEDA computed with the dispersion-corrected PBE-D2 functional (28.6 kcal/mol, Table 3) was significantly higher than the one computed by the PBE functional without dispersion interactions (5.6 kcal/mol, [23]). Actually, previous studies on metal organic precursors on silica slabs have also evidenced an important role of dispersion interactions on the physisorption process [31].

In a more general scenario, the present quantitative results on the energy barrier for the metal-ligand bond dissociation step underline that at temperatures close to the conditions adopted in CVD processes, the thermal energy (kT) is comparable with the bond stretching energies (at T = 500 K, kT ≈ 1 kcal/mol), and it can be of the same order of magnitude of the activation barrier, as found for Zn(hfa)_2_TMEDA. Consequently, thermal effects may significantly influence the precursor chemical behavior in the first key stages of vapor deposition processes and affect the whole decomposition mechanism.

In this context, further insight could be gained by comparing the thermal behavior of the Zn complex with that predicted by our previous AIMD studies on the Fe [24] and Cu [22] homologues. In all these cases, a high-temperature-induced rolling diffusion of the metal precursor took place on the substrate surface, accompanied by large-amplitude oscillations of metal-ligand bond distances. In the case of Fe(hfa)_2_TMEDA, the oscillations of Fe-N bonds were larger with respect to Fe-O ones: In particular, one of the Fe-N distances averaged to 4.52 ± 0.73 Å over the trajectory, indicating that the rolling motion caused a partial TMEDA detachment already in the early simulation stages. This behavior finally led to a TMEDA ligand loss of the iron complex (as reported in Ref. [24]) with formation of the tetra-coordinated Fe(hfa)_2_ moiety.

On the other hand, in the case of the Zn complex, the oscillations of Zn-O bonds were larger than those of Zn-N distances, as evidenced in Figure 1, Table 1, and in [23]. Additionally, here we have shown that the Zn complex could isomerize to a penta-coordinated pyramidal structure stabilized by hydrogen-bonding with a surface hydroxyl group, and that the process barrier was of the same order of magnitude of the thermal energy available in the system at the deposition conditions (500–750 K). Finally, even the diffusional rolling motion of the Cu(hfa)_2_TMEDA precursors on a hydroxylated silica surface led preferentially to larger-amplitude Cu-O oscillations [22], suggesting that Cu-O bond dissociation might be favored over the Cu-N one. On this basis, it would be reasonable to expect that a similar octahedral-to-pyramidal conversion could take place even for Cu(hfa)_2_TMEDA.

Finally, an interesting question is whether the thermal behavior and bond dissociation mechanism deduced for Zn(hfa)_2_TMEDA on hydroxylated silica might be extended to other kinds of substrates—for example, electrically conductive tin dioxide and other transparent oxidic supports used for ZnO deposition. Whereas the metal oxide nature may undoubtedly influence the activation barrier, it is worthwhile highlighting that the presence of surface hydroxyl groups is rather ubiquitous on many oxide-based materials (see, e.g., [73,74,75]). Hence, it can be argued that the hydrogen bond-assisted formation of the square-pyramidal complex reported in our work might be quite general and not limited to hydroxylated silica. 

## 5. Conclusions

This study has elucidated the metal-ligand bond dissociation process of a β-diketonate-diamine Zn precursor for the CVD of zinc oxide nanomaterials. An octahedral-to-pyramidal isomerization of the Zn(hfa)_2_TMEDA complex involving a Zn-O bond dissociation can be triggered by the rolling diffusion of this molecule on a heated hydroxylated silica surface, which causes the partial detachment of an hfa ligand. The free energy barrier calculated for the octahedral-to-pyramidal conversion is of the same order of magnitude of thermal energy at 500 K. Stabilization of the activated complex, which exhibits a trigonal-bipyramidal geometry, is provided by strong hydrogen bonding interactions between the uncoordinated hfa oxygen and a surface hydroxyl group. This finding clearly highlights that under CVD conditions, activation mechanisms very different from those typical of (room temperature) chemistry may be viable, thus yielding species obtainable only under high-temperature conditions. Comparison of the thermal behavior of Zn(hfa)_2_TMEDA with previously studied homologues suggests that even the copper homologue may exhibit a similar isomerization to a pyramidal form in contact with hydroxylated oxide surfaces. Interesting future perspectives for the development of the present research activities might concern the investigation of the fate of this family of precursors on other kinds of substrates or in the presence of oxygen.

Overall, this work highlights the role of rolling molecular diffusion in surface processes typical of vapor deposition fabrication routes of oxide nanomaterials. The octahedral-to-pyramidal conversion unraveled here may be crucial for the subsequent reactive event, since it may enable the metal center to come into direct contact with other reagents on the substrate surface.

## Figures and Tables

**Table 1:** Average metal-ligand bond distances **<r>**, standard deviation **σ**, minimum **r_min_** and maximum **r_max_** distance values sampled from the AIMD simulation of Zn(hfa)_2_TMEDA on the hydroxylated SiO_2_ surface at the PBE-D2/PW level. All distances are in Å. For atom labeling, see Figure 2.

Distance	<r>	σ	r_min_	r_max_
Zn-O_1_	2.23	0.25	1.82	3.24
Zn-O_1′_	2.04	0.12	1.75	2.43
Zn-O_2_	2.15	0.21	1.82	2.89
Zn-O_2′_	3.62	0.91	1.86	5.21
Zn-N	2.26	0.18	1.85	2.93
Zn-N′	2.33	0.19	1.94	2.89

**Table 2:** Geometrical parameters calculated in the gas phase at 0 K for octahedral (Oct) and pyramidal (Pyr) forms of Zn(hfa)_2_TMEDA at PBE-D2/PW and M06/D95+(d,p) levels (labels in Figure 2). Distances in Å, angles in degrees (°).

Parameter	Oct- PBE-D2/PW	Pyr- PBE-D2/PW	Oct- M06/D95+(d,p)	Pyr- M06/D95+(d,p)
Zn-O_1_	2.115	2.057	2.074	2.031
Zn-O_1′_	2.116	2.064	2.097	2.039
Zn-O_2_	2.115	1.964	2.074	1.942
Zn-O_2′_	2.116	4.334	2.097	4.302
Zn-N	2.196	2.188	2.162	2.152
Zn-N’	2.196	2.167	2.162	2.123
O_2′_-C_2′_	1.269	1.241	1.251	1.225
O_2_-C_2_	1.270	1.282	1.254	1.267
O_1′_-C_1′_	1.269	1.271	1.251	1.254
O_1_- C_1_	1.270	1.273	1.254	1.256
C_2_*-C_2′_	1.408	1.437	1.404	1.437
C_2_*-C_2_	1.405	1.387	1.400	1.378
C_1_*-C_1′_	1.408	1.405	1.404	1.399
C_1_*-C_1_	1.405	1.404	1.400	1.400
O_2_-Zn-O_1_	88.0	111.9	87.3	111.8
O_2_-Zn-O_1′_	86.7	108.1	86.6	107.0
O_2_-Zn-N	93.8	99.3	94.2	99.8
O_2_-Zn-N’	91.6	91.5	92.0	92.2

**Table 3:** Free energy differences ΔG and energy differences ΔE for the transition state and Pyr with respect to Oct. Binding energies BE (kcal/mol) of the octahedral (Oct) and pyramidal (Pyr) structures of the Zn complex adsorbed on the SiO_2_ silica slab. Binding energy difference Δ(BE) relative to Oct.

(kcal/mol)	Oct@SiO_2_	TS@SiO_2_	Pyr@SiO_2_
**ΔG**	0	+5.80	+3.57
**ΔE**	0	+7.80	+2.23
**BE**	28.27	-	36.07
Δ(BE)	0	-	7.81

**Table 4:** Geometrical parameters of the octahedral (Oct), pyramidal (Pyr), and transition state (TS) structures of the Zn complex adsorbed on the hydroxylated SiO_2_ surface at the PBE-D2/PW level. Isolated complex data at the same theory level are reported to facilitate comparison. Distances in Å.

Distance	Oct@SiO_2_	TS@SiO_2_	Pyr@SiO_2_	Isolated-Oct	Isolated-Pyr
Zn-O_1_	2.087	2.090	2.044	2.115	2.057
Zn-O_1′_	2.139	2.018	2.091	2.116	2.064
Zn-O_2_	2.115	2.042	2.050	2.115	1.964
Zn-O_2′_	2.263	3.818	4.873	2.116	4.334
Zn-N	2.177	2.141	2.160	2.196	2.188
Zn-N’	2.149	2.145	2.147	2.196	2.167
O_2′_-H*	1.885	1.630	1.586	-	-
O_2′_-C_2′_	1.279	1.248	1.251	1.269	1.241
O_2_-C_2_	1.264	1.265	1.277	1.270	1.282
O_1′_-C_1′_	1.267	1.277	1.269	1.269	1.271
O_1_- C_1_	1.271	1.269	1.272	1.270	1.273

H* indicates the surface hydroxyl proton involved in the O_2′_-H* hydrogen bond.

## Data Availability

Data is contained within the article or supplementary material.

## Supplementary Materials

The following are available online. Movie S1: movie from the AIMD PBE-D2/PW trajectory evidencing the rolling diffusion of Zn(hfa)_2_TMEDA.

## Sample Availability

Samples of the compounds M(hfa)_2_TMEDA (M = Fe, Cu, Zn) are available from the authors.
